# Material Sensing with Spatial and Spectral Resolution Based on an Integrated Near-Infrared Spectral Sensor and a CMOS Camera

**DOI:** 10.3390/s25113295

**Published:** 2025-05-23

**Authors:** Ben Delaney, Sjors Buntinx, Don M. J. van Elst, Anne van Klinken, René P. J. van Veldhoven, Andrea Fiore

**Affiliations:** Department of Applied Physics, Eindhoven Hendrik Casimir Institute, Eindhoven University of Technology, P.O. Box 513NL, 5600 MB Eindhoven, The Netherlands

**Keywords:** spectral sensing, material classification, near infrared, integrated photonics

## Abstract

Measuring the composition of materials at a distance is a key requirement in industrial process monitoring, recycling, precision agriculture, and environmental monitoring. Spectral imaging in the visible or near-infrared (NIR) spectral bands provides a potential solution by combining spatial and spectral information, and its application has seen significant growth over recent decades. Low-cost solutions for visible multispectral imaging (MSI) have been developed due to the widespread availability of silicon detectors, which are sensitive in this spectral region. In contrast, development in the NIR has been slower, primarily due to the high cost of indium gallium arsenide (InGaAs) detector arrays required for imaging. This work aims to bridge this gap by introducing a standoff material sensing concept which combines spatial and spectral resolution without the hardware requirements of traditional spectral imaging systems. It combines spatial imaging in the visible range with a CMOS camera and NIR spectral measurement at selected points of the scene using an NIR spectral sensor. This allows the chemical characterization of different objects of interest in a scene without acquiring a full spectral image. We showcase its application in plastic classification, a key functionality in sorting and recycling systems. The system demonstrated the capability to classify visually identical plastics of different types in a standoff measurement configuration and to produce spectral measurements at up to 100 points in a scene.

## 1. Introduction

Multispectral imaging (MSI) and hyperspectral imaging (HSI) have emerged as a powerful technique for material identification and analysis, leveraging their ability to capture detailed spectral information over the different parts of a scene [1]. Over the past two decades, advancements in M/HSI technology have broadened their application spectrum, ranging from environmental monitoring and agriculture to medical diagnostics and industrial inspection [2,3,4]. Indeed, spectral imaging provides detailed insights into the chemical composition and physical properties of different objects and materials in a scene, in a stand-off configuration [5,6].

Multispectral imaging captures fewer, broader bands with lower spectral resolution, making it simpler, cheaper, and suitable for broad applications. Hyperspectral imaging, on the other hand, captures many narrow, contiguous bands with high spectral resolution, enabling detailed analysis but requiring more complex and costly processing. Despite its limited spectral resolution, multispectral imaging can be sufficient for many tasks, such as vegetation analysis and remote sensing.

In the visible range, silicon-based detectors have facilitated the development of cost-effective MSI systems due to their sensitivity and widespread availability. These systems have found applications, for example, in precision agriculture, where they are used for crop monitoring and disease detection [7], and in environmental studies for mapping vegetation and assessing water quality [8]. In contrast, the NIR region, which covers wavelengths from approximately 700 nm to 2500 nm, offers unique advantages for analyzing materials that are indistinguishable in the visible spectrum. NIR spectral sensing and imaging is particularly effective in identifying organic compounds, making it invaluable in industries such as pharmaceuticals, food safety, and recycling. For instance, NIR spectral measurements can detect moisture content in food [9], assess the authenticity of pharmaceutical products [10], analyze forensic traces [11], and sort different types of plastics in recycling facilities [12].

Despite its advantages, the adoption of NIR his has been hampered by the high cost and complexity of the required technology. CMOS sensors can only cover a spectral range up to 1000–1100 nm—for example, the Sony IMX174 is sensitive up to approximately 1000 nm, and the Sionyx XQE-1350 and XQE-1351 sensors have extended sensitivity up to around 1100 nm [13]. For accessing the NIR range beyond 1100 nm, indium gallium arsenide (InGaAs) detector arrays are needed, which are significantly more expensive than silicon detectors. Both linear InGaAs arrays, used for whiskbroom HIS, [5] and 2D arrays, used for pushbroom and snapshot MSI [5], feature many pixels, corresponding to large chip area and complex readout electronics. This cost barrier has limited the accessibility and widespread use of NIR spectral imaging, particularly in industries where budget constraints are a critical consideration.

The objective of this study is to address the cost and accessibility challenges associated with NIR spectral imaging by developing a low-cost NIR sensing system with spatial and spectral resolution. To this aim, we note that, *in most applications, acquiring spectral information in each point of a scene is not required*—in reality, composition information is needed for a limited number of objects, which can be identified with simple visible imaging. For example, the objects of interest may be the plastics pieces being moved along a sorting line or leaves and fruit in a greenhouse. In these situations, an alternative strategy is the use of a visible CMOS camera for the identification of the points of interest and the subsequent spectral measurement at these points using scanning optics and a single-point spectrometer or spectral sensor. In order to avoid the cost and large footprint of NIR spectrometers, we propose the use of an integrated InGaAs multipixel spectral sensor [14,15] for the spectral measurement. Indeed, these NIR spectral sensors have been shown to provide sufficient spectral information for material quantification and classification purposes. The system demonstrated in this work integrates a CMOS camera, scanning optics, and a fiber-coupled 14-pixel detector array sensitive in the 850–1700 nm region, where fiber coupling is used to scramble unwanted spatial information in the spectral measurement. To demonstrate the effectiveness of the proposed spatial/spectral sensing system, we selected plastic sorting as application example. The recycling industry faces a critical need for rapid, low-cost identification and sorting of plastics to enhance recycling efficiency and reduce environmental impact. Color cameras and visual inspection often struggle to distinguish between different plastic types, while the NIR spectral signatures allow accurate differentiation. Our system’s ability to scan and identify different plastic samples highlights its potential for plastic sorting but also for process and quality control in agricultural and industrial production.

## 2. Materials and Methods

**Figure 1:**
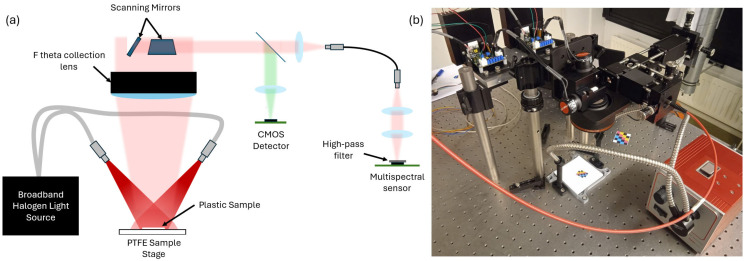
(**a**) Schematic diagram of the experimental setup; (**b**) photograph of the experimental setup.

The proposed system integrates several components to combine imaging in the visible spectrum and spectral sensing in the NIR. These components include galvanometer-based scanning mirrors, a multispectral sensor, a standard CMOS camera (Logitech C270, Logitech International S.A., Lausanne, Switzerland), and a halogen light source (Thorlabs OSL2IR, Thorlabs GmbH, Bergkirchen, Germany). A schematic of the experimental system is shown in Figure 1.

### 2.1. Multispectral Sensors

**Figure 2:**
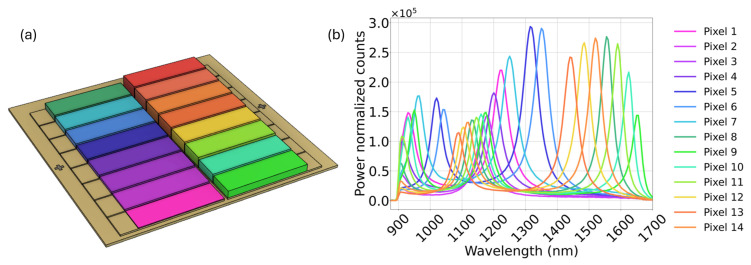
(**a**) Illustration of the multispectral sensor used for the experiments contained in this paper. (**b**) Power-normalized multispectral sensor spectral response.

The multispectral sensor at the core of the system has been developed in our group in recent years [14,15], and it is commercially available [16] (multispectral sensors in the visible range and up to 1000 nm, based on silicon detector arrays with integrated filters, are available from, for example, ams OSRAM [17]). It is based on an array of sixteen resonant-cavity-enhanced photodetectors, which are sensitive between 900 nm and 1700 nm [14]. Figure 2a shows a rendering of the multispectral sensor, while Figure 2b presents the spectral responsivity of the sensor used in this work. While the detector in use was intended to function as a sixteen-pixel device, two of the pixels have wire bonding defects. Thus, they are omitted from all the data which are presented as well as all model training and validation datasets.

Multiple quantification and classification applications of this multispectral sensor have been shown to date, including plastic classification, the quantification of moisture level in rice, narcotic classification, and the quantification of fat content in milk [14]. While the sensor has been shown to perform nearly as accurately as conventional grating spectrometers for classification and quantification, all the published measurements were based on a configuration where the sample is in contact or close vicinity to the sensor. In this work, we show its application in a stand-off configuration, in combination with imaging and scanning, enabling a much wider application space in standoff and remote sensing. The linewidth of the 14 pixels varies in the range (55 ± 7) nm. Information on the fabrication of the sensor can be found in the work of van Klinken et al. [15].

### 2.2. Sensing System

The field of view of the system is uniformly illuminated from two sides by a halogen light source (Thorlabs OSL2IR), which emits between 400 nm and 1800 nm, with a power density, integrated across the spectrum, of 1.5 W/cm^2^. An illumination angle of 60° is chosen, to minimize specular reflection in the collection path.

The diffuse reflection from the illuminated sample is collected through an F-theta lens, by the scanning mirrors. The working distance of the F-theta lens is 284 mm. The two scanning mirrors can be rotated independently, allowing the placement of the collection spot (image of the fiber) at a specific position within the field of view of the system. The collected light is then passed through a dichroic mirror, which separates the light into visible (VIS) and NIR components, directing them through two separate optical paths. The samples are placed on a white PTFE sample stage.

The NIR light is coupled into a 500 um-core multimode optical fiber using a lens with a 25 mm focal length. The light transmitted by the fiber is then collected and focused onto the multispectral sensor by a pair of 25 mm focal length lenses. An 850 nm high-pass filter (Thorlabs FELH0850, Thorlabs GmbH, Bergkirchen, Germany) is placed in front of the multispectral sensor to block visible light. The use of a fiber was found to be useful to avoid spatial imaging of the sample onto the multispectral array, whereby a spatial intensity distribution on the sensor is erroneously interpreted as spectral information. The multimode fiber acts a very efficient diffuser, scrambling spatial information and allowing a correct spectral measurement on the collection spot. An alternative free-space coupling to the sensor, using a ground glass diffuser (Thorlabs DG10-600, Thorlabs GmbH, Bergkirchen, Germany), was found to provide reduced sensing performance.

The visible light reflected by the dichroic mirror is focused onto the detector of a CMOS webcam. The area captured in the visible image includes the area the multispectral sensor is measuring, as well as an area around it. The diameter of the visible image and the diameter of the collection area are 1.2 cm and 3.5 mm, respectively. The field of view for the experiments is 5 × 5 cm^2^. The collection and imaging area can be chosen independently with a proper choice of optics. The camera allows identifying the regions of interest where spectral measurements must be performed

The outputs from both the NIR multispectral sensor and the CMOS camera can be captured for a given position of the scanning mirror, or, alternatively, the system can be operated as a point scan spectral imager by scanning and capturing spectral data across a specified field of view.

While this is not further described here, it is straightforward to add additional spectral channels, by splitting the beam in free space or with a fiber splitter. The resulting beams are then coupled (via filters if needed) to spectral sensors optimized for different spectral ranges (e.g., one sensor for the visible range and one for the NIR). This system allows obtaining discrete measurements or a full multispectral image over the combined spectral range, a feature not available in pushbroom or snapshot spectral imaging systems.

### 2.3. Measurement Protocol

Four different types of plastic were employed in this work: polypropylene (PP), polyethylene terephthalate (PET), high-density polyethylene (HDPE), and low-density polyethylene (LDPE). The samples were larger than the collection area of the spectral sensor, 1.2 cm diameter, to ensure repeatable characterization. The signal-to-noise ratio (SNR) averaged across all fourteen active pixels for measurement of the white reference background is ~1100, achieved with an exposure time of 75 ms, with five exposures taken and averaged for each measurement. A dark measurement, where the illumination source is powered off, is also captured with the same exposure settings and averaged and subtracted from all measurements. This dark subtraction is necessary for robust classification but only needs to be carried out at the start of an experiment as the system is otherwise very stable with fluctuations on the dark signal of less than 10 counts, with a measured signal of 1000 counts.

Multispectral data were captured for both a training and test set as follows. The training set consisted of 6 PP, 5 PET, 5 HDPE, and 5 LDPE samples, with multispectral data being captured for each sample at 6 positions across the field of view of the system.

**Figure A1:**
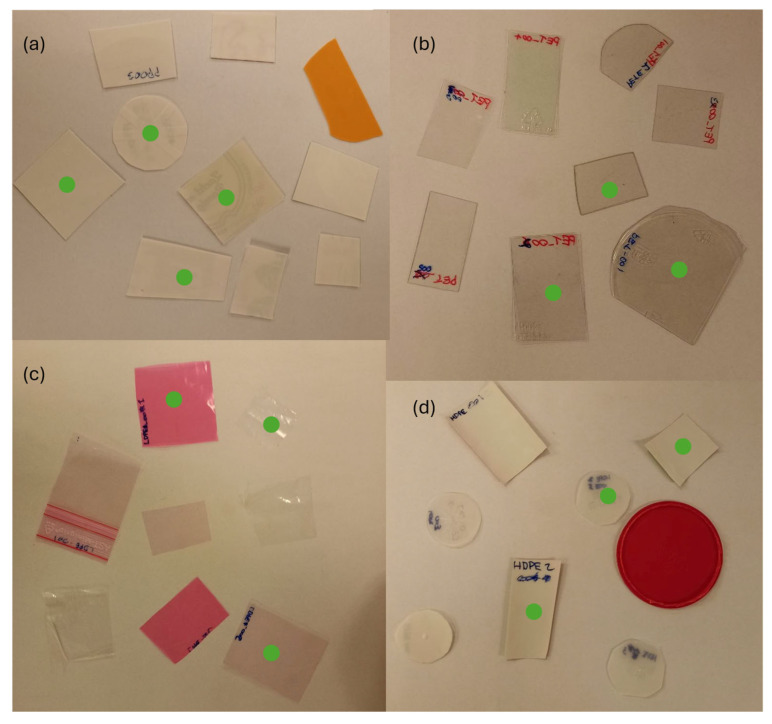
Photograph showing the appearance of samples measured for the plastic classification experiment. (**a**) PET; (**b**) PET (**c**) LDPE; (**d**) HDPE. The green circles indicate the samples that were used in the test set.

The test data set consisted of 4 PP, 3 PET, 3 HDPE, and 3 LDPE samples, with multispectral data being collected for each sample at 4 positions across an area bound by the area enclosed by the locations where the training data were captured. The samples were measured in the order listed at every measurement location for both the training and testing dataset. The training and test samples were completely independent and captured on separate days. Figure A1 in Appendix A shows the plastic samples used.

For all measurements, the plastic samples were positioned randomly, and the scanning mirrors were moved to the angle corresponding to the desired viewing position of the system. Each sample was then placed with its center at the point of measurement, and 5 measurements were acquired for every sample. This methodology was used for all samples at every measurement position.

### 2.4. Classification Model Building

The plastic classification model is built using a robust workflow tailored for processing multispectral data to classify various types of plastics. This workflow leverages partial least squares discriminant analysis (PLS-DA) for its classification.

To prepare the data for model training, samples are aggregated, and each is labelled according to its respective material type. The aggregated data undergo a normalization process to ensure consistency across samples. Specifically, sum normalization is applied, where the photocurrent value for each pixel is divided by sum of all pixel photocurrents. This normalization step standardizes the input features, effectively mitigating the impact of intensity fluctuations in the experimental system.

The code then employs cross-validation to determine the optimal number of latent variables for the PLS-DA model. Cross-validation involves partitioning the data into several folds and iteratively training and validating the model to assess its performance. This process identifies the number of latent variables that yield the highest accuracy.

Once the optimal number of latent variables is determined, the model is trained on the entire training dataset, without any folding. The trained model’s performance is evaluated using accuracy scores and confusion matrices, which provide insights into the classification accuracy and the distribution of correctly and incorrectly classified samples.

All data analysis and visualization algorithms were implemented in Python 3.11 (Python Software Foundation, Beaverton, OR, USA) using packages from NumPy 1.24.3 [18], Matplotlib 3.7.1 [19], Seaborn 0.12.2 [20], and Scikit-learn 1.3.0 [21].

## 3. Results

Examples of the multispectral data used in the training and validation datasets as well as confusion matrices generated by the constructed model are given in Section 3.1. To demonstrate the viability of using the presented system for collecting spatial and spectral information, an experiment which captures multispectral and imaging data simultaneously is carried out. The scanning mirrors are used to raster over a portion of the FOV of the system in which three different plastic samples are placed. A reconstructed image of the raster image is presented, along with classification labels in Section 3.2.

### 3.1. Model Building and Classification Verification

**Figure 3:**
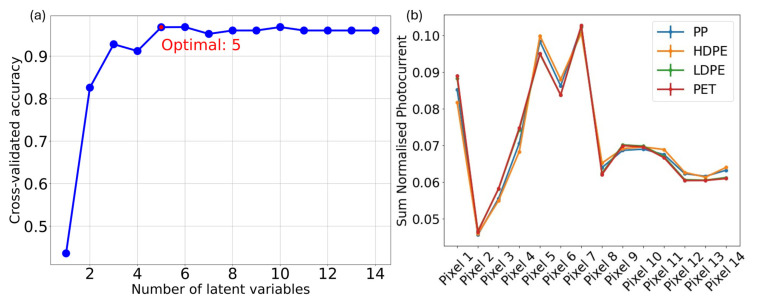
(**a**) Example multispectral sensor output. (**b**) Variation of cross-validated accuracy with number of latent variables, showing the optimal number of latent variables for the trained model worked in this work.

Figure 3a shows an example comparison of a multispectral measurement at the same measurement position for the four different investigated plastic types. There is a clear variation between pixel values for the different sample classes. After sum-normalization, the variation in measurements for each plastic type at different positions is minimal, and the corresponding standard deviation is not larger than the standard deviation for acquisitions at the same position. Collection through a fiber was found to be critical to achieve this condition.

The data acquired for the training set of samples were fed to the model building code. The initial cross-validation yielded the highest accuracy with five latent variables, which can be seen in Figure 3b. The relatively small differences between the curves in this figure are expected and consistent with the performance of our device across various classification/quantification problems. Despite their visual similarity, these variations are sufficient to enable effective feature separation, as demonstrated by the performance of the classification model.

**Figure 4:**
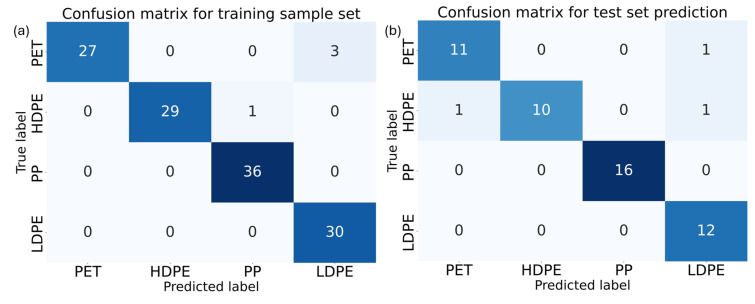
(**a**) Aggregate cross validated matrix of the training data. (**b**) Confusion matrix for the validation sample set tested against the model built on the training data.

Therefore, the classification model was built using five latent variables, as shown in Figure 4a. The validation sample was tested with this classification model, which resulted in the confusion matrix in Figure 4b. These confusion matrices illustrate the effectiveness of the classification model in identifying different types of plastics, specifically PET, HDPE, PP, and LDPE.

In the aggregated confusion matrix for the prediction model, which represents the training dataset, the model shows strong performance. For PET, 27 samples were correctly identified, while only 3 were misclassified as LDPE, indicating a high level of accuracy for PET classification, though there is some overlap in distinguishing PET from LDPE. HDPE classification is also highly accurate, with 29 out of 30 samples correctly identified, and only one misclassification occurring as PP. The model demonstrates excellent precision in classifying PP, with all 36 samples correctly identified, reflecting its robustness in identifying this plastic type. Similarly, LDPE is perfectly classified across all 30 samples.

When examining the confusion matrix for the test sample set, the high classification accuracy is confirmed. PET samples are correctly classified 11 out of 12 times, with one instance of misclassification as LDPE. HDPE shows a slight decrease in performance compared to the training set, with 10 correct classifications out of 12; misclassifications occur with one sample each being incorrectly identified as PET and LDPE. Despite these minor errors, the model maintains a strong overall accuracy. For PP, the model’s performance remains flawless, as all 16 samples in the test set are correctly classified, confirming its reliability in identifying this plastic type. LDPE also sees perfect classification, with all 12 samples correctly identified, reflecting consistent accuracy.

Overall, the model demonstrates a high level of accuracy (94% for the test set used), particularly with PP and LDPE, which are classified without error in both the training and test datasets. There are minor misclassifications between PET and LDPE, which may be due to these materials exhibiting very similar spectral absorption profiles in the NIR region, particularly when the samples are thin and provide minimal diffuse reflection. As a result, in some edge cases, the model may misclassify one material for another due to the high degree of spectral overlap, but the overall performance suggests that the model is robust and effective for plastic classification tasks.

### 3.2. Spectral Sensing at Multiple Spots in a Scene

While, in the previous measurements, a single object was present in a field of view, here we assess the system’s ability to provide spatial and spectral information over a complex scene, which represents the basic functionality of spectral imaging systems. To this aim, three samples of plastic are placed on the sample plane. The scanning mirrors move in the raster pattern over a portion of the sample plane, which is broken into a 10 × 10 grid; at each point on the grid, an image is captured; and the multispectral sensor measurement is acquired. Each multispectral sensor measurement is captured with the same integration time as used to build the model in Section 3.1 and is the average of five measurements.

**Figure 5:**
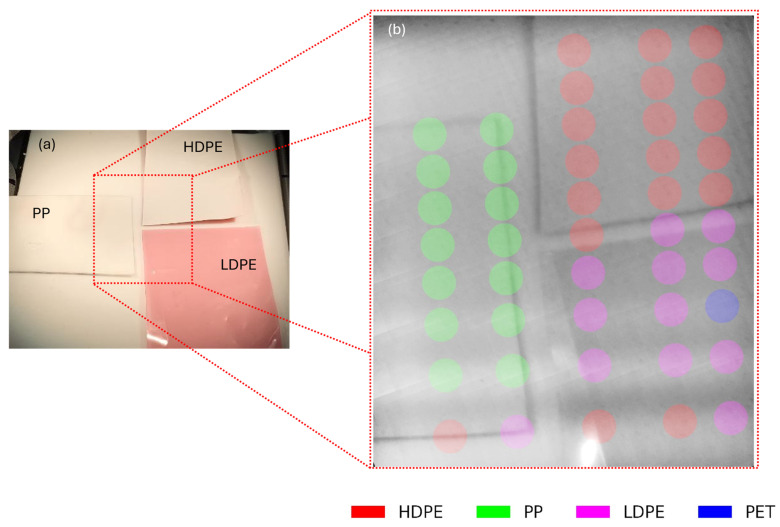
Spectral sensing in a complex scene. (**a**) Camera image of plastic samples on sample plane. (**b**) Reconstructed image from raster images, with circles in false color indicating the classification result at that point. The circle represents the projected area of the multispectral sensor on the image plane.

The multispectral data are fed into the model generated in Section 3.1, which classifies the sample at every point from which the multispectral sensor acquired. The images from each point are combined to visualize the sample plane in a full field image. This full field image is then combined with the sample classifications to generate a multispectral image. An example of this spatially resolved spectral sensing is shown in Figure 5, part (a) shows an off-axis camera image of the sample plane, while part (b) shows a reconstructed image, with the false-color circles denoting the classification of the sample at those points. Although 100 spectral measurements were taken, only 50 are shown for image clarity.

This measurement shows that the system can mostly classify PP, HPDE, and LDPE. As expected, the classification fails in some cases along the edge of the samples and also if there is a large direct reflection at the point of measurement.

## 4. Discussion

This work advances the field of NIR MSI by demonstrating that cost-effective solutions can be developed without sacrificing performance. The system’s ability to identify and classify plastics underscores its relevance in recycling industries, where rapid, low-cost sorting is essential to improve sustainability and reduce environmental impact. Moreover, the standoff configuration expands the application space of the sensor array, enabling remote sensing and analysis for diverse industrial and environmental scenarios. Beyond recycling, this approach could be transformative for industries such as food quality monitoring, pharmaceuticals, and agriculture.

While the system achieves high accuracy overall, it exhibits limitations in specific scenarios. Misclassifications along the edges of samples suggest sensitivity to spatial heterogeneity or optical artifacts. This issue could be mitigated by enhancing the spatial resolution of the detector array. Although this was not implemented in the present system, it is envisaged that machine vision can be used to automatically identify where in the field of view an item of interest is, align the collection spot with the item of interest, and measure, thus eliminating the issue at edges of samples.

Strong specular reflections disrupt spectral measurements, leading to misclassification. This is a common issue in NIR diffuse reflection techniques for which a viable hardware solution has yet to be found. Handling this issue in software is a non-ideal but workable solution. Misclassification of the background as LDPE highlights the need for improved preprocessing and model refinement, particularly adding measurements of the sample plane, without a sample, to the training and test dataset, or by performing outlier analysis.

While the current measurement speed is limited by the readout of the multispectral sensor (~1.2 s per measurement spot), this could be reduced by an order of magnitude with improved electronics. The scanning system could also be implemented, alternatively, on a gantry in a core xy configuration, for example; this might be more suitable in industrial environments due to the elimination of delicate scanning mirrors. Through the use of additional optics, e.g., dichroic mirrors, it would also be possible to split the collected light and add other spectral sensors which are sensitive in other wavelength regions, allowing for simultaneous measurements.

## 5. Conclusions

In this article, we demonstrate the possibility of integrating imaging and spectral sensing with an array of resonant-cavity-enhanced photodetectors into a standoff measurement. The ability of this standoff measurement system to classify plastic samples has been shown, as well as the ability to provide spectral sensing data throughout a scene, which, with different optical configurations, could be integrated into industrial production line platforms. This is a significant step for this type of sensor, extending its potential application to non-contact measurements at a distance, while using an artificial NIR light source. Other possible applications of standoff and spatially resolved spectral sensing could be found in agrifood to monitor produce in greenhouses, as well as in the health sector for the classification of tumors. While this work focuses on use cases with artificial illumination, it may be possible to implement the device in drone applications, if the sensing problem is compatible with a lower signal-to-noise ratio. The possible application of this method to measuring film thicknesses across a wafer area in semiconductor metrology can also be investigated.

We have demonstrated the integration of a multispectral sensor into a classification system which does not require dispersive optics or high-cost detectors, which are used as the standard in current scientific and industrial applications. This novel approach lowers the barrier to entry for robust material classification in the 1000 nm–1700 nm region.

## Data Availability

The data which support the findings of this study are available from the corresponding author upon reasonable request.
